# Geranylgeraniol Restores Zoledronic Acid-Induced Efferocytosis Inhibition in Bisphosphonate-Related Osteonecrosis of the Jaw

**DOI:** 10.3389/fcell.2021.770899

**Published:** 2021-11-03

**Authors:** Xin Chen, Weiwen Zhu, Rongyao Xu, Xin Shen, Yu Fu, Jie Cheng, Laikui Liu, Hongbing Jiang

**Affiliations:** ^1^Jiangsu Province Key Laboratory of Oral Diseases, Nanjing Medical University, Nanjing, China; ^2^Department of Oral and Maxillofacial Surgery, Affiliated Hospital of Stomatology, Nanjing Medical University, Nanjing, China; ^3^Jiangsu Province Engineering Research Center of Stomatological Translational Medicine, Nanjing Medical University, Nanjing, China; ^4^Department of Stomatology, Jiangyin People’s Hospital, Wuxi, China; ^5^Department of Basic Science of Stomatology, The Affiliated Stomatological Hospital of Nanjing Medical University, Nanjing, China

**Keywords:** bisphosphonate-related osteonecrosis of the jaw, macrophages, phagocytosis, apoptosis, zoledronic acid

## Abstract

Bisphosphonate-related osteonecrosis of the jaw (BRONJ) is a severe side effect of long-term administration of bisphosphonates such as zoledronic acid (ZA), but its pathogenesis remains unclear. Impairment of the clearance of apoptotic cells (termed “efferocytosis”) by ZA may be associated with the pathogenesis of BRONJ. The aim of this study was to investigate whether ZA might inhibit macrophage efferocytosis and promote osteocytic apoptosis, and the underlying mechanisms responsible for the disturbing balance between clean and generation of osteocytic apoptosis. We found that ZA significantly promoted the apoptosis of osteocyte and pre-osteoblast via BRONJ mouse models and *in vitro* MC3T3-E1 but also inhibited the efferocytosis of macrophage on apoptotic cells. Moreover, supplement with geranylgeraniol (GGOH), a substrate analog for geranylgeranylation of Rac1, could restore Rac1 homeostasis and rescue macrophage efferocytosis. GGOH partially inhibits MC3T3-E1 apoptosis induced by ZA via downregulation of Rac1/JNK pathway. We also examined the Rac1 distribution and activation conditions in bone marrow-derived macrophages (BMDMs) and MC3T3-E1 under ZA treatment, and we found that ZA impaired Rac1 migration to BMDM membrane, leading to round appearance with less pseudopodia and efferocytosis inhibition. Moreover, ZA simultaneously activated Rac1, causing overexpression of P-JNK and cleaved caspase 3 in MC3T3-E1. Finally, the systemic administration of GGOH decreased the osteocytic apoptosis and improved the bone healing of the extraction sockets in BRONJ mouse models. Taken together, our findings provided a new insight and experimental basis for the application of GGOH in the treatment of BRONJ.

## Introduction

Bisphosphonate-related osteonecrosis of the jaw (BRONJ) is one serious complication of long-term use of bisphosphonates, such as zoledronic acid (ZA) (Singh and Gonegandla, 2020). The accepted pathogenesis of BRONJ includes osteoclast dysfunction, microvascular embolization, and emerging immune disorders (Hoefert et al., 2016; Patntirapong and Poolgesorn, 2018; Yu and Su, 2020). Nevertheless, the therapy available for this condition is challenging.

Approximately one billion apoptotic cells (ACs) are produced in an adult human every day (Boada-Romero et al., 2020; Doran et al., 2020). However, these cells can be cleaned up with very high efficiency to prevent accumulation of dead cells, secondary necrosis, and tissue inflammation (Boada-Romero et al., 2020). The clearance of ACs, termed “efferocytosis,” is one of the most important functions of professional phagocytes including macrophages (Zheng et al., 2021). Macrophages could specifically recognize and contact “eat me” signal on the surface of ACs (Penberthy and Ravichandran, 2016; Gordon and Plüddemann, 2018). The engagement of cell-surface receptors on macrophages activates the Rho superfamily such as Rac1 and downstream actin-related protein 2/3 (ARP2/3) complex, which then polymerizes actin to form the phagocytic cup and internalize the ACs (Gordon and Plüddemann, 2018; Arienti et al., 2019). Empty bone lacunas with osteocytic apoptosis were frequently observed in BRONJ models, indicating that the balance between the generation and clearance of ACs in bone has been broken by bisphosphonates (Kikuiri et al., 2010; Aguirre et al., 2012). We thus suspect that increased ACs might be an important mediator involved in BRONJ development, which could be attributed to impaired macrophage efferocytosis and accumulated bisphosphonate-induced osteocytic ACs. Furthermore, the underlying mechanism needs to be fully elucidated.

Rac1, one of mammalian Rho GTPases, is implicated in regulation of membrane ruffling, cytoskeletal rearrangement, migration, and apoptosis (Nobes and Hall, 1995; Mattila and Lappalainen, 2008). Before performing the proper function, Rac1 needs prenylation modification for anchoring to plasm membranes and interaction with downstream signaling effectors (Gao et al., 2009). However, ZA proves to inhibit FPP synthase, which is essential for the synthesis of geranylgeranyl pyrophosphate (GGPP; the substrate used for Rac1 posttranslational prenylation) (Dunford et al., 2006). Previous studies showed that exogenous geranylgeraniol (GGOH) is converted into GGPP in the mevalonate pathway and antagonizes the side effects of ZA on osteoblasts, osteoclasts, and gingival fibroblasts *in vitro* (Nagaoka et al., 2015; Fliefel et al., 2019; Patntirapong et al., 2021) and the development of BRONJ *in vivo* (Nagaoka et al., 2015; Koneski et al., 2018). However, the influence of GGOH on macrophage efferocytosis and ACs in extraction sockets is still unknown.

In the present study, we specifically focused on the correlations between ACs and the development of BRONJ. We aimed to investigate whether GGOH can neutralize the negative effects of ZA in terms of osteocytic apoptosis and macrophage efferocytosis, which may provide a new insight for the treatment of BRONJ.

## Materials and Methods

### Generation of Bisphosphonate-Related Osteonecrosis of the Jaw-Like Mouse Model

BRONJ-like models in mice were established based on our described methods (Zhu et al., 2019). Briefly, male C57BL/6J mice at 4 weeks of age were intraperitoneally injected with ZA (250 μg/kg) twice a week. One week after ZA treatment, the right first maxillary molars were extracted under deep anesthesia via the intraperitoneal injection of ketamine (100 mg/kg). In other experiments, GGOH (250 μg/kg, Sigma-Aldrich Corp., St. Louis, MO, United States) was simultaneously injected twice a week with ZA after tooth extraction. The vehicle group was treated with equivalent phosphate-buffered saline (PBS). After 4 weeks, the maxillary bones were collected for histologic evaluation, microcomputed tomography analysis, and TUNEL assay.

The osteonecrotic area in extraction sockets was used for sample determination. A total of 15 mice with five for each group were needed to detect a difference of 20% in osteonecrotic area with a standard deviation of 5% at a significance level of 0.05 and a power of at least 80%. The mice were allocated into the cages, with five animals housed per standard cage at 22–25°C with unlimited rodent chow and water by block randomization. No specific inclusion and exclusion criteria were required. The mouse procedures were conducted in accordance with the ARRIVE (Animal Research: Reporting of *in vivo* Experiments) guidelines and approved by the Committee of Nanjing Medical University for Animal Resources (#1805006).

### Micro-Computed Tomography Analysis

Images were obtained using a micro-CT device (SkyScan 1176; Bruker, Kontich, Belgium). The maxillae were scanned at high resolution (18 μm) with the energy of 50 kV and 456 μA. Bone mineral density (BMD) and bone volume/tissue volume (BV/TV) were evaluated using CTAN v.1.13.8.1 software (SkyScan).

### Histologic and Immunofluorescence Staining

The bone specimens were prepared in 4-μm-thick paraffin-embedded sections for hematoxylin and eosin (H&E) staining. The empty osteocyte lacuna percentage was counted in at least three slices of the tooth extraction per mouse to calculate the necrotic bone area. The detection of ACs in formalin-fixed paraffin sections was performed using the cell apoptosis detection kit (Vazyme, Nanjing, China).

### Bone Marrow-Derived Macrophage Culture

Bone marrow-derived macrophage (BMDM) culture was performed as before (Zhu et al., 2019). Briefly, bone marrow cells isolated from the tibias of 5- to 8-week-old male C57BL/6 mice were initially incubated for 4 h. Then the non-adherent cells were collected and continuously cultured in complete Dulbecco’s modified Eagle medium (DMEM) as well as murine macrophage colony-stimulating factor (M-CSF; 10 ng/ml) for 3 days. The fresh medium was added on day 3, and the cells were cultured for another 4 days. The mature macrophages were harvested and used for subsequent experiments.

### Detection of Active Rac1

MC3T3-E1 cells were treated with PBS, ZA, and Rac1 inhibitor (NSC 23766, HY-15723A, Sigma) for 24 h. The activation of Rac1 was assessed by Rac activation assay kit (NewEast Biosciences, King of Prussia, PA, United States). Briefly, cells were lysed on ice for 15 min. After centrifugation at 12,000 × *g* for 10 min at 4°C, the supernatant was mixed with anti-active Rac1 monoclonal antibody and protein A/G agarose bead slurry for 1 h at 4°C. Then the bead-bound GTP-Rac1 was resuspended with 2 × sodium dodecyl sulfate–polyacrylamide gel electrophoresis (SDS-PAGE) sample buffer and analyzed by Western blotting.

### Annexin V/Propidium Iodide Dual Staining Assay

MC3T3 cells were treated with ZA and GGOH of various concentrations for 24 or 48 h. Cells were then collected and stained with Alexa Fluor 488 Annexin V (dilution 1:20) and propidium iodide (PI) (dilution 1:1,000) for 15 min. The analysis was performed using a FACSVerse flow cytometer (BD Biosciences, San Jose, CA, United States).

### Efferocytosis Assay

For induction of apoptosis, cell medium was removed before UV irradiation and replaced with PBS. MC3T3-E1 or H9 cells were irradiated (covers opened) at room temperature with 254-nm UV generated by the biosafety cabinet (SG403aHE, BAKER, Sanford, ME, United States) for 45 min (Widel et al., 2014). The cells were cultured in a CO_2_ incubator for another 4 h. The non-adherent cells were then collected and analyzed by flow cytometry. That the Annexin V-positive and PI-negative cell proportion was above 80% was regarded as the effective induced apoptosis.

To label the apoptotic MC3T3-E1 or H9 cells, cells were incubated with 1 μM of carboxyfluorescein succinimidyl ester (CFSE) at 37°C for 10 min and then at 4°C for another 5 min at a cell concentration of 10^7^ cells/ml. After being washed twice with PBS, cells were resuspended with corresponding complete medium.

For efferocytosis capacity detection, BMDMs were seeded to plates at 10^6^ cell/well and cocultured with ZA (0, 1, 10, and 20 μM) as well as GGOH (0, 3, 6, and 10 μM) for 24 h. The medium containing 10^7^ apoptotic H9 or 5 × 10^6^ apoptotic MC3T3-E1 was added and cocultured for 1 or 2 h. After being rinsed with PBS, BMDMs were incubated with 2 μl of Rat Anti-Mouse F4/80 (T45-2342, BD Pharmingen, San Diego, CA, United States) at 37°C for 10 min and washed three times with cold PBS. Cells were then fixed with 4% paraformaldehyde for 30 min at room temperature. The proportion of phagocytic BMDMs was evaluated using microscopy or by flow cytometry (Kourtzelis et al., 2019; Zhang et al., 2019). Under microscopy, BMDMs are red, and apoptotic MC3T3-E1s are green. The mix of red and green is yellow, showing the BMDMs containing ACs.

### Statistical Analysis

The data were analyzed and recorded as means ± SEM using Prism 5 software (GraphPad Software, La Jolla, CA, United States). Comparisons between the two groups were analyzed using the two-tailed, unpaired Student’s t-test. One-way ANOVA followed by Tukey’s multiple comparisons was adopted to analyze the difference among three or more groups. A value of *p* < 0.05 was considered statistically significant.

## Results

### Zoledronic Acid Inhibits Macrophage Efferocytosis and Induces Pre-osteoblast Cell Apoptosis

We initially established models of BRONJ-like disease in wild-type (WT) mice. TUNEL staining of the maxillary interalveolar septum on the non-extracted side showed that ZA could significantly increase ACs concentrating in bone marrow and alveolar margins (Supplementary Figure 1). The *in vivo* finding indicated that the balance between the generation and clearance of ACs in BRONJ mouse models had been broken.

**FIGURE 1:**
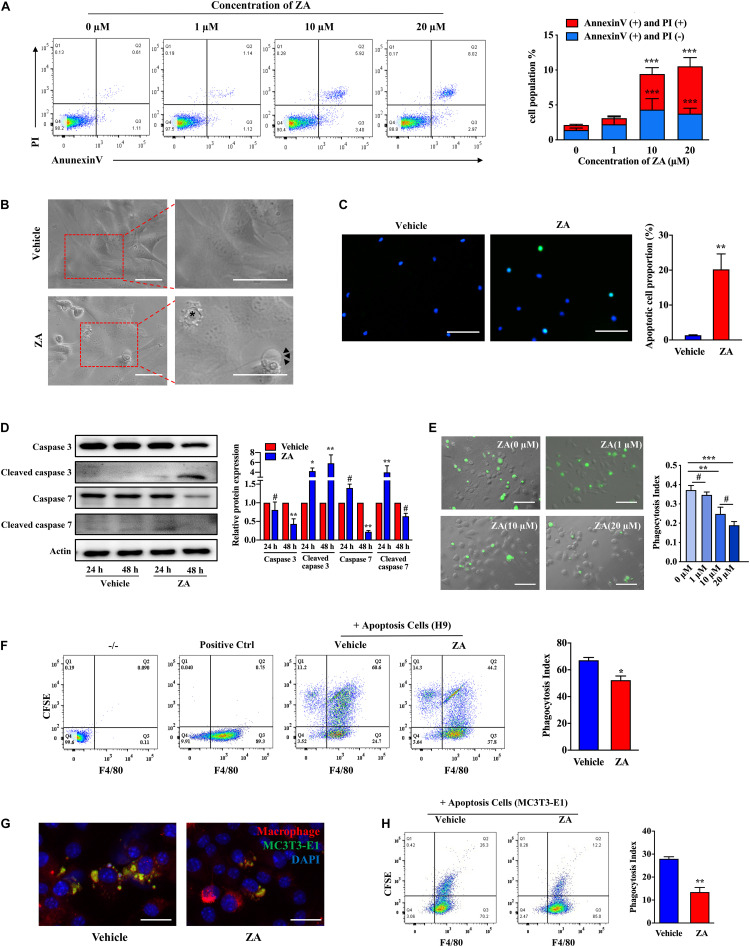
ZA promotes the MC3T3-E1 apoptosis and impairs the efferocytosis of macrophages *in vitro*. **(A)** MC3T3-E1 were treated with ZA of various concentrations for 24 h. Cells were stained with Annexin V and PI and then subjected to flow cytometry. **(B)** Bright-field images of MC3T3-E1 treated with ZA. Apoptotic cells are indicated by an asterisk. Features include plasma membrane blebbing. Cells experiencing secondary necrosis are marked with arrows, characteristic of plasma membrane extrusions. Scale bar, 50 μm. **(C)** TUNEL images of MC3T3-E1 treated with ZA. Scale bar, 100 μM. **(D)** Protein expression of total caspase 3, cleaved caspase 3, total caspase 7, and cleaved caspase 7 in MC3T3-E1 treated with ZA for 24 or 48 h. **(E)** Representative macroscopic imaging of macrophage efferocytosis. CFSE-labeled H9 cells and the ZA-treated BMDMs were cocultured for 1 h. Relative efferocytosis index is shown and was calculated as the ratio of macrophages that have phagocytosed apoptotic H9 to the total number of macrophages. Scale bar, 100 μm. **(F)** Flow cytometry analysis of macrophage phagocytosis of CFSE-labeled apoptotic H9 cells. Representative plot shows cells double positive for CFSE and the macrophage marker F4/80 indicating efferocytosis. **(G)** Representative images of BMDMs (F4/80, red) ingested apoptotic MC3T3-E1 (CFSE, green) for 2 h. BMDMs were stimulated with vehicle or ZA (10 μM) for 24 h before addition of CFSE-labeled apoptotic MC3T3-E1. Scale bar, 50 μm. **(H)** Flow cytometry analysis of macrophage phagocytosis of CFSE-labeled apoptotic MC3T3-E1 cells. Representative plot shows cells double positive for CFSE and the macrophage marker F4/80 indicating efferocytosis. The data are presented as the mean ± SEM values (*n* = 3). *p < 0.05, **p < 0.01, ***p < 0.001, **^#^***p* > 0.05. ZA, zoledronic acid; PI, propidium iodide; CFSE, carboxyfluorescein succinimidyl ester; BMDMs, bone marrow-derived macrophages.

We then investigated the effect of ZA on cell apoptosis *in vitro*. Flow cytometry analysis showed that the apoptosis of MC3T3-E1 could be easily induced by ZA in a concentration-dependent manner (Figure 1A). MC3T3-E1 showed obvious fibroblast like morphology with a round nucleus, continuous cell membrane, and clear boundary (Figure 1B). When treated with ZA for 24 h, cells showed apoptotic appearance characterized by dense small bubbles on the plasma membrane. Secondary necrotic features, such as plasma membrane protrusions, were also detected under the light microscope (Figure 1B). MC3T3-E1 treated with ZA displayed significantly elevated numbers of cells with positive TUNEL staining as compared with MC3T3-E1 treated with vehicle (Figure 1C). Furthermore, ZA treatment resulted in MC3T3-E1 apoptosis as observed by Western blotting analysis of increased cleaved caspase 3 and cleaved caspase 7 at 24 h (Figure 1D).

We next examined macrophage efferocytosis activity, a key element in clearance of ACs in bone tissue. After coculture with apoptotic H9 for 1 h, phagocytic BMDMs were decreased in the medium- (10 μM) or high-concentration (20 μM) ZA group compared with the control group (*p* < 0.01) (Figure 1E). However, no significant difference was observed between high-concentration and medium-concentration groups (*p* > 0.05). We thus selected medium concentration of ZA for further efferocytosis assay. Flow cytometry directly showed that the proportion of phagocytotic BMDMs was reduced under ZA treatment (*p* < 0.05) (Figure 1F). After that, we cocultured BMDMs with apoptotic MC3T3-E1 and found that BMDMs could hardly capture ACs after ZA treatment (Figure 1G). Flow cytometry analysis also showed that phagocytotic BMDMs in the control group were much more than those in ZA-treatment group (*p* < 0.01) (Figure 1H). These results indicate that ZA induces osteocytic apoptosis and inhibits the clearance of ACs.

### Geranylgeraniol Could Improve Zoledronic Acid-Inhibited Macrophage Efferocytosis

**FIGURE 2:**
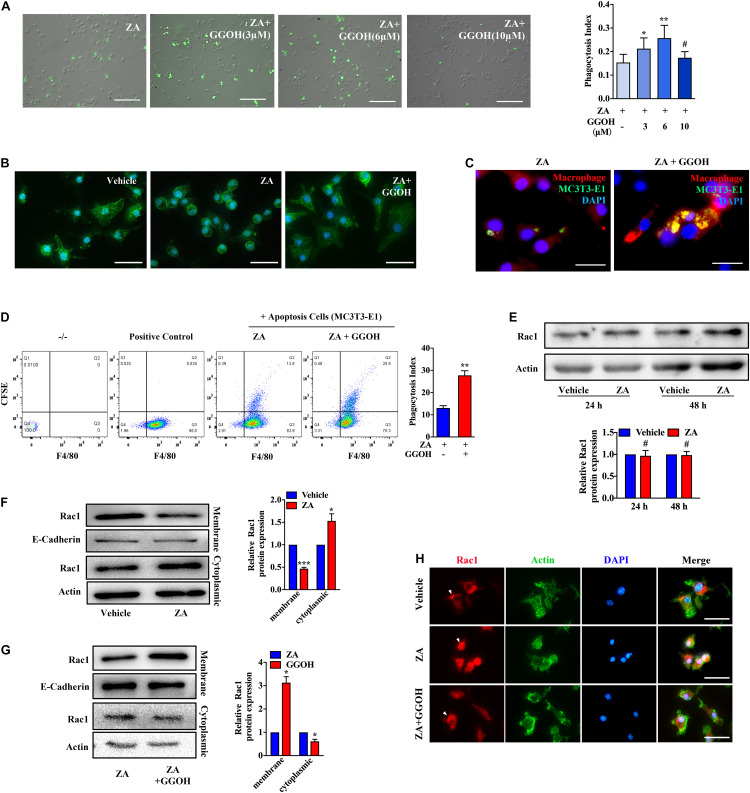
GGOH restores ZA-induced inhibition of Rac1 membrane displacement and macrophage efferocytosis. **(A)** After treatment with ZA for 24 h, BMDMs were subjected to CFSE-labeled apoptotic H9 for 1 h. The comparison was performed with ZA group. Scale bar, 200 μm. **(B)** Morphology of BMDMs treated with vehicle, ZA, and GGOH for 24 h. Scale bar, 100 μm. **(C)** Representative images of BMDMs (F4/80, red) ingested apoptotic MC3T3-E1 (CFSE, green). Scale bar, 50 μm. **(D)** Flow cytometry analysis of macrophage phagocytosis of CFSE-labeled apoptotic MC3T3-E1 cells. Representative plot shows cells double positive for CFSE and the macrophage marker F4/80 indicating efferocytosis. **(E)** BMDMs were treated with 10 μM ZA or vehicle for 24 or 48 h. The total Rac1 expression was detected using Western blotting. **(F)** Cytoplasmic and membrane Rac1 were separated and detected in BMDMs following ZA treatment for 24 h. **(G)** The cytoplasmic and membrane Rac1 were detected in BMDMs treated with ZA or ZA + GGOH. **(H)** Representative fluorescence micrographs of BMDMs stained for Rac1, Actin, and nuclear marker with following treatments. Scale bar, 100 μm. The data are presented as the mean ± SEM values (n = 3). *p < 0.05, **p < 0.01, ***p < 0.001, **^#^***p* > 0.05. GGOH, geranylgeraniol; ZA, zoledronic acid; BMDMs, bone marrow-derived macrophages; CFSE, carboxyfluorescein succinimidyl ester.

GGOH, an analog of isoprene substrate, was adopted to observe its rescue effect on macrophage efferocytosis impaired by ZA. The numbers of BMDMs containing H9 increased significantly in the ZA + GGOH (3 μM) and ZA + GGOH (6 μM) groups (*p* < 0.05), but not in the ZA + GGOH (10 μM) group (*p* > 0.05) (Figure 2A). Furthermore, the phagocytosis index of ZA + GGOH (6 μM) group was obviously larger than that of ZA + GGOH (3 μM) group. We thus selected 6 μM of GGOH in following efferocytosis assay. Round appearance with fewer pseudopodia following ZA treatment was observed (Figure 2B). Interestingly, BMDMs restored elongation appearance with increased pseudopodia cocultured with exogenous GGOH (Figure 2B). As expected, GGOH promoted macrophage to capture more apoptotic MC3T3-E1 under ZA treatment (Figure 2C). Flow cytometry analysis also showed that the phagocytotic BMDMs in the ZA + GGOH group were much more than those in the ZA group (*p* < 0.01) (Figure 2D).

It has been reported that membrane Rac1 serves an important function in modulating cytoskeletal rearrangement and efferocytosis. Taking into consideration the key function of Rac1 protein in efferocytosis, its cellular expression was measured using Western blotting. No change was found in total Rac1 expression in ZA-treated BMDMs (Figure 2E). However, Rac1 appeared more in the cytoplasm and less on the membrane after ZA treatment (Figure 2F). The supplement with GGOH could significantly increase Rac1 expression on cell membrane and decrease Rac1 expression in cytoplasm compared with that in the ZA group (Figure 2G). The fluorescence staining showed that the vehicle group presented with dominant Rac1 expression on the membrane, whereas ZA-treated cells showed notable cytoplasmic Rac1. The addition of GGOH could rescue Rac1 distribution to the membrane (Figure 2H). Since there is no isoprene-related Rac1 antibody available, we chose its analog, unprenylated-Rap1a antibody, to indirectly detect the isoprene of Rho GTPases in BMDMs. ZA could significantly increase the level of unprenylated-Rap1a in BMDMs in a concentration-dependent manner (Supplementary Figure 2). These results imply that ZA might impair cellular Rac1 distribution through inhibition of Rac1 isoprene and that GGOH may improve ZA-inhibited macrophage efferocytosis via restoring Rac1 membrane translocation.

### Geranylgeraniol Partially Inhibits MC3T3-E1 Apoptosis Induced by Zoledronic Acid

**FIGURE 3:**
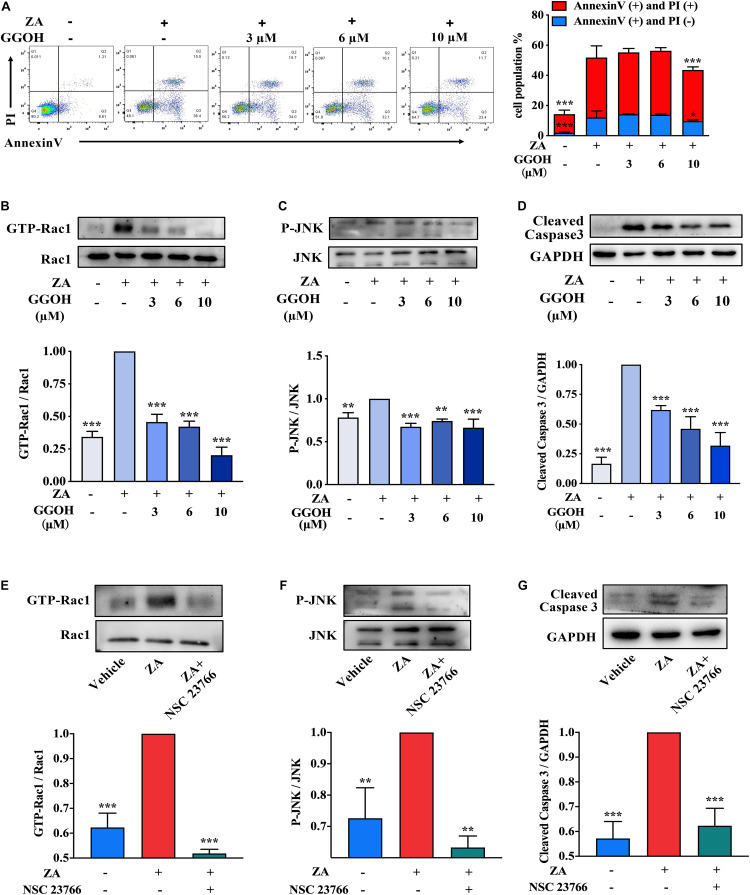
GGOH could reduce ZA-induced apoptosis of MC3T3-E1 via inhibiting Rac1 overactivation. **(A)** MC3T3-E1 were cultured with ZA (10 μM) in the presence or absence of GGOH (0–10 μM) for 24 h. Cells were stained with Annexin V and PI and subjected to flow cytometry. Comparison of apoptosis was performed with ZA group. **(B–D)** MC3T3-E1 were cultured with ZA (10 μM) in the presence or absence of GGOH (0–10 μM) for 24 h. The expression levels of active Rac1 (GTP-Rac1), active JNK (P-JNK), and cleaved caspase 3 were measured by Western blotting. **(E–G)** MC3T3-E1 were cultured with a vehicle, ZA (10 μM) in the presence or absence of NSC 23766. The expression levels of active Rac1 (GTP-Rac1), active JNK (P-JNK), and cleaved caspase 3 were measured by Western blotting. The data are presented as the mean ± SEM values (*n* = 3). *p < 0.05, **p < 0.01, ***p < 0.001. GGOH, geranylgeraniol; ZA, zoledronic acid; PI, propidium iodide.

Flow cytometry analysis was performed to evaluate the apoptotic rate in MC3T3-E1 treated with ZA as well as GGOH of various concentrations. Supplement of GGOH (10 μM) significantly reduced the AC proportion compared with the ZA group (Figure 3A). GGOH could reduce the expression of GTP-Rac1, P-JNK, and cleaved caspase 3 in a concentration-dependent manner (Figures 3B–D). Western blotting data indicated that ZA resulted in increased GTP-Rac1 and P-JNK expression, while GTP-Rac1 downregulation resulted in decreased P-JNK and cleaved caspase 3 levels (Figures 3E–G). These data demonstrate that GGOH could inhibit overactivation of Rac1 to reduce MC3T3-E1 apoptosis caused by ZA treatment.

### Geranylgeraniol Partially Reduces Apoptotic Cell in Extraction Socket and Restores Bone Healing in Bisphosphonate-Related Osteonecrosis of the Jaw-Like Mice

**FIGURE 4:**
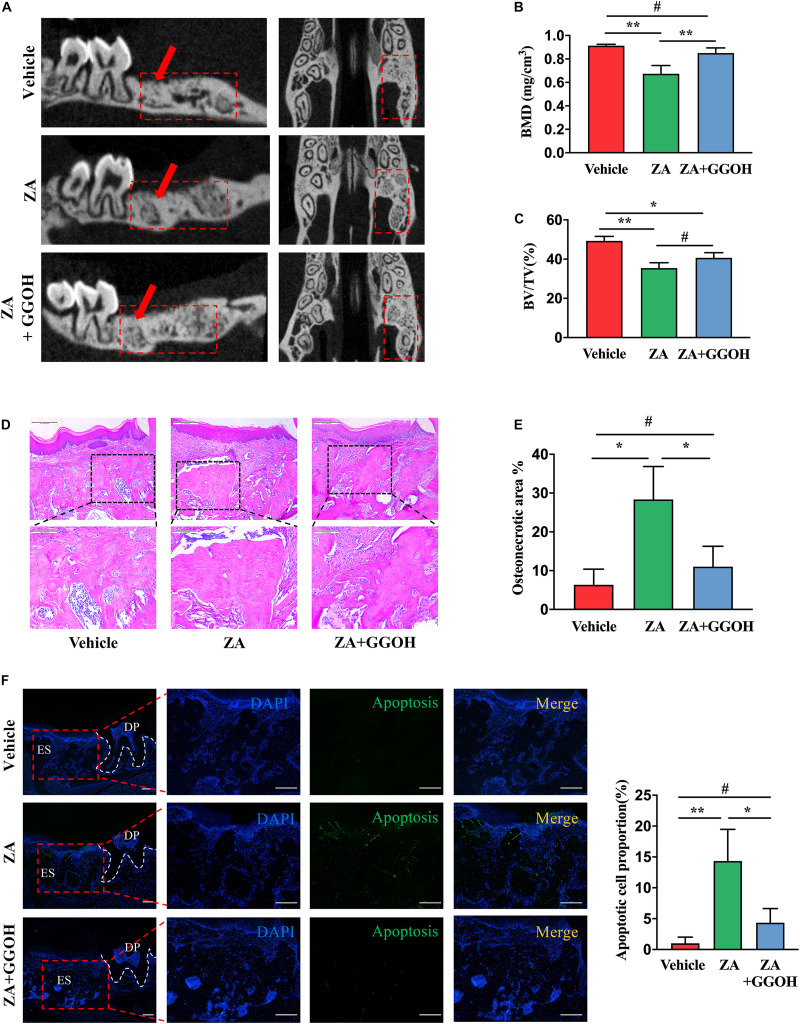
GGOH prevents the development of BRONJ. **(A–C)** Micro-CT analysis of the first molar distal buccal root was performed. **(D,E)** H&E staining of the extraction socket 4 weeks after tooth extraction. Scale bar, 500 μm. **(F)** TUNEL staining the extraction socket 4 weeks after tooth extraction. Scale bar, 100 μm. ES, extraction socket; DP, dental pulp. The data are presented as the mean ± SEM values (*n* = 3). *p < 0.05, **p < 0.01, **^#^***p* > 0.05. GGOH, geranylgeraniol; BRONJ, bisphosphonate-related osteonecrosis of the jaw.

BRONJ-like lesions with empty osteocytic lacunae in extraction sockets were observed after ZA treatment. New woven bone formation was significantly increased in the ZA + GGOH group in comparison with that in the ZA group (Figures 4A–C). Histologically, the vehicle group showed socket filling with woven bone and a pronounced periosteum at the alveolar crest (Figure 4D). In contrast, the ZA group showed impaired bone healing and osteonecrotic areas with empty osteocyte lacunae. A marked inflammatory infiltrate was noted adjacent to the osteonecrotic area. The ZA + GGOH group demonstrated filling with woven bone with few scattered empty osteocyte lacunae. The percentage of osteonecrotic area was significantly decreased in the ZA + GGOH group compared with that in the ZA group (Figure 4E). Apoptosis could hardly be captured in vehicle sockets (Figure 4F). The prominent ACs concentrated on alveolar crest were observed in extraction sockets after ZA treatment. In contrast, simultaneous injection with GGOH could reduce ACs to a normal level (Figure 4F). All these findings suggest that GGOH rescues the ZA-impaired socket healing and represses osteocytic apoptosis.

## Discussion

**FIGURE 5:**
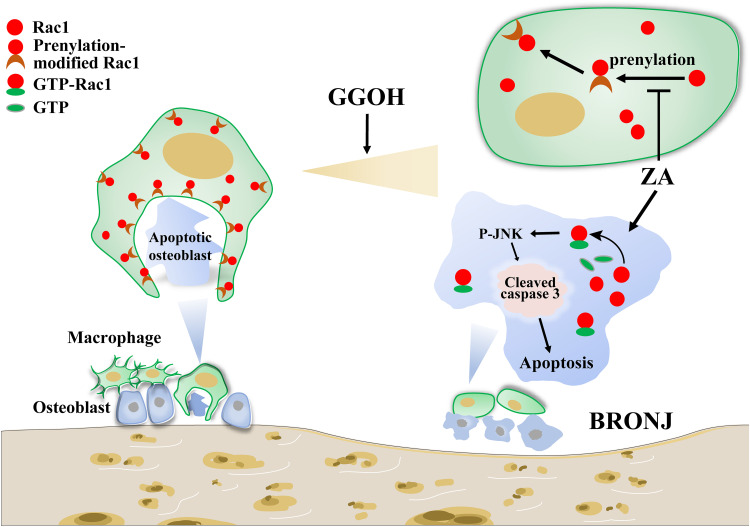
Working model of ZA in inducing osteocytic apoptosis of BRONJ. On the one hand, ZA could overactivate Rac1 to stimulate JNK phosphorylation, leading to cell apoptosis. On the other hand, ZA inhibits Rac1 membrane translocation, causing difficulty in macrophage morphological deformation and efferocytosis. Supplement of GGOH, a neutralization drug against ZA-disturbed Rac1 status, could restore macrophage efferocytosis and prevent osteocytic apoptosis in the development of BRONJ. ZA, zoledronic acid; BRONJ, bisphosphonate-related osteonecrosis of the jaw.

In the present study, we demonstrated that the induction of BRONJ-like disease in mice might be attributable to imbalance between generation and clearness of osteocytic apoptosis. Briefly, for osteocytes, ZA overactivates Rac1 signaling and induces cleaved caspase 3 overexpression, therefore leading to increased osteocytic apoptosis. For macrophages, ZA proves to inhibit Rac1 prenylation and disturb the membrane distribution of cellular Rac1, resulting in deformity of macrophage morphology and efferocytosis impairment. Our findings suggest that supplement of GGOH, a neutralization drug against ZA-disturbed Rac1 status, could restore macrophage efferocytosis and prevent osteocytic apoptosis in the development of BRONJ (Figure 5).

As a common cause of BRONJ, extraction accompanies numerous mechanical and thermal damage, resulting in osteocytic apoptosis and death (Schwarze et al., 2021). Macrophages immediately reach the socket to clean up the debris and apoptosis cells. Additionally, macrophages recruit and differentiate bone-marrow mesenchymal stem cells through efferocytosis. If efferocytosis is forbidden, these ACs would experience secondary necrosis, leading to autoimmunity, tissue necrosis, and pathological inflammation (Doran et al., 2020).

Recent studies have revealed an essential role of macrophages in the development of BRONJ (Zhu et al., 2019; Yang et al., 2020; Zhao et al., 2020). We previously showed that macrophages orchestrate inflammation trends by shifting their phenotypes in microenvironment, in which efferocytosis is necessary for its anti-inflammatory response (Zhu et al., 2019). Furthermore, ZA was reported to alter macrophage morphology and reduce macrophage phagocytosis of particles of zymosan fluorescein conjugate (Hoefert et al., 2016). We selected adherent MC3T3-E1 and suspension H9 as phagocytic targets to further verify the ZA impaired macrophage efferocytosis. Small GTPases of the Rho superfamily, such as Rac1, CDC42, and RhoA, are linked to the regulation of actin cytoskeleton. However, only heterozygous deletion of gene Rac1, but not Cdc42 and RhoA, could reverse inflammation in mouse models for arthritis, indicating the special role of Rac1 in chronic inflammation diseases (Akula et al., 2019). As a membrane protein, Rac1 needs to rearrange distribution and interact with downstream signaling effectors to form phagocytotic cup when encountering ACs (Tao et al., 2015; Pan et al., 2019). We suggest that ZA might impair macrophage efferocytosis via inhibiting Rac1 prenylation. Supplement of exogenous GGOH could be converted to GGPP and then drive the geranylgeranylated Rac1 production, which is depleted by ZA or alendronate (Dunford et al., 2006). Our study showed that GGOH could restore the impaired macrophage efferocytosis by ZA. GGOH also proves to increase cell viability and differentiation of osteoblast precursors, osteoclasts, and vascular smooth muscle cells under bisphosphonate treatment (Zafar et al., 2016; Fliefel et al., 2019; Irwin et al., 2020). Taken together, these findings all imply GGOH as a potential target for BRONJ treatment.

Macrophages always form canopy structure over mature osteoblasts at sites of bone formation (Pazianas, 2011). In addition, cell-to-cell contact results in the differentiation and maturation of bone marrow-derived mesenchymal stem cells via oncostatin M and bone morphogenetic protein 2 production by macrophage (Pazianas, 2011; Pajarinen et al., 2019; Sims and Martin, 2020). Furthermore, approximately 95% of the bone surface is covered with osteoblast-related cells (Seeman and Martin, 2019; Sims and Martin, 2020). We thus selected MC3T3-E1 as the proper target for efferocytosis and apoptosis analysis *in vitro* without identification of the specific ACs in sections of BRONJ mouse model. As showed by a previous study, ZA treatment induced MC3T3-E1 apoptosis in a concentration-dependent manner (Patntirapong et al., 2021). ZA treatment triggered sustained activation of Rac1, and Rac1-induced p38 activation could partially suppress the pro-apoptotic effect in osteoblast-like cells (Dunford et al., 2006). However, several other studies have reported contradictory results, suggesting that active Rac1 induces apoptosis in various cell lines. Jin et al. revealed that Rac1 is active during tumor necrosis factor-α (TNF-α)-induced apoptosis in intestinal epithelial cells and that the inhibition of Rac1 substantially prevents apoptosis via the TNF-α-induced activation of JNK (Jin et al., 2006). In a recent study, inhibition of miR-224 increased the apoptotic level of dental pulp stem cells, in addition to the expression levels of active Rac1 and P-JNK, while Rac1 silencing restored the apoptosis and expression of the P-JNK to normal levels (Qiao et al., 2020). Our study showed that ZA increased GTP-Rac1 and P-JNK expression in MC3T3-E1, while Rac1 inhibitor decreased P-JNK and cleaved caspase 3 levels. This suggests that Rac1/JNK pathway might participate in the apoptosis triggered by ZA.

GGOH is reported to reduce the side effects of nitrogen-containing bisphosphonates on bone generation (Nagaoka et al., 2015; Koneski et al., 2018). Treatment with GGOH restored the ZA-inhibited number of TRAP-positive multinuclear cells derived from mouse osteoclast precursors and neutralized the inhibitory effects of ZA on osteoclastogenesis and bone remolding (Nagaoka et al., 2015). The present study provides more basic evidence for supporting GGOH as a potential approach for BRONJ treatment. Furthermore, a recent study indicated that GGOH may have a positive or negative effect on the differentiation of osteoblasts dependent on the concentration (Patntirapong et al., 2021). Researchers have showed that GGOH could rescue the cell viability and biological function up to a certain limit (Fliefel et al., 2019). At lower concentrations, it had an antagonistic effect to ZA. However, at higher concentrations, GGOH had enhanced the cell death. From the available *in vitro* studies, the concentration ratio of GGOH and ZA should preferably not exceed 100% (Nagaoka et al., 2015; Fliefel et al., 2019; Patntirapong et al., 2021). As shown in the present study, GGOH of high concentration (10 μM) could not improve macrophage efferocytosis reduced by ZA, suggesting that an optimum amount of GGOH is necessary for BRONJ treatment. So far, there are only two *in vivo* studies available evaluating the influence of GGOH on the development of BRONJ (Nagaoka et al., 2015; Koneski et al., 2018). One study utilized a probe for medical delivery to extraction sockets in mice without specifying the value of GGOH (Koneski et al., 2018). We thus adopted the systemic administration of GGOH as the other study did (Nagaoka et al., 2015). Nevertheless, topical GGOH should be tested to promote better extraction socket healing and prevent BRONJ in further studies.

In summary, to the best of our knowledge, we have demonstrated for the first time that GGOH could neutralize the negative effects of ZA in terms of osteocytic apoptosis and macrophage efferocytosis. By utilizing GGOH, we presented a novel therapy for improvement of Rac1 status and treatment of BRONJ. More experiments are required to elucidate the therapeutic and safe potential of GGOH on BRONJ.

## Data Availability Statement

The raw data supporting the conclusions of this article will be made available by the authors, without undue reservation.

## Ethics Statement

The animal study was reviewed and approved by The animal study was reviewed and approved by the animal Ethics Committee of Nanjing Medical University (#1805006).

## Author Contributions

XC contributed to the conception, design, data acquisition, and interpretation and drafted and critically revised the manuscript. WZ contributed to the conception, design, data acquisition, and interpretation and critically revised the manuscript. RX contributed to the conception and design and revised the manuscript. XS contributed to the conception, design, and data acquisition. YF contributed to the conception and design and drafted and critically revised the manuscript. JC and LL contributed to the critical revision of the manuscript. HJ contributed to the conception and design and critically revised the manuscript. All authors gave their final approval and agreed to be accountable for all aspects of the work.

## Conflict of Interest

The authors declare that the research was conducted in the absence of any commercial or financial relationships that could be construed as a potential conflict of interest.

## Publisher’s Note

All claims expressed in this article are solely those of the authors and do not necessarily represent those of their affiliated organizations, or those of the publisher, the editors and the reviewers. Any product that may be evaluated in this article, or claim that may be made by its manufacturer, is not guaranteed or endorsed by the publisher.
