# Supplementary material for: Geranylgeraniol Restores Zoledronic Acid-Induced Efferocytosis Inhibition in Bisphosphonate-Related Osteonecrosis of the Jaw
Source: Front Cell Dev Biol. 2021 Nov 3;9:770899. doi: 10.3389/fcell.2021.770899 (PMC8595285; doi:10.3389/fcell.2021.770899)
Supplement: Supplementary file 1 [file Data_Sheet_1.docx]

Supplementary Material

# Supplementary Figures

## Supplementary Figures


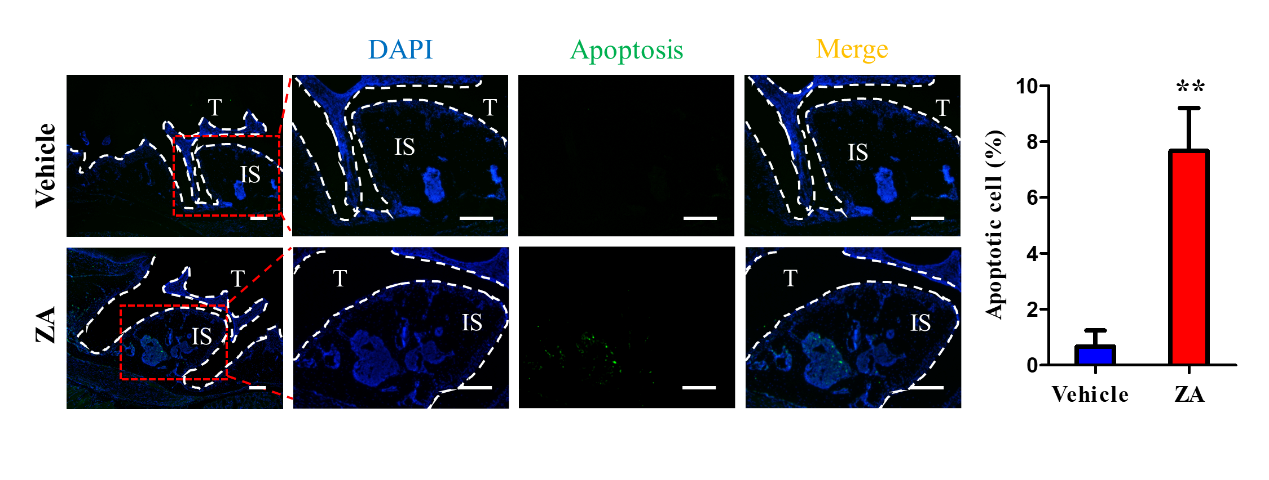


**Supplementary Figure 1.** ZA induces cellular apoptosis in bone tissue *in vivo*.

TUNEL staining of maxilla from WT mice treated with ZA (250 µg/kg) and vehicle for 5 weeks. Photos were taken at the interalveolar space between the first maxillary molars and the scale bar of 100 µm was added. T: tooth; IS: interalveolar space. *******P*<0.01.


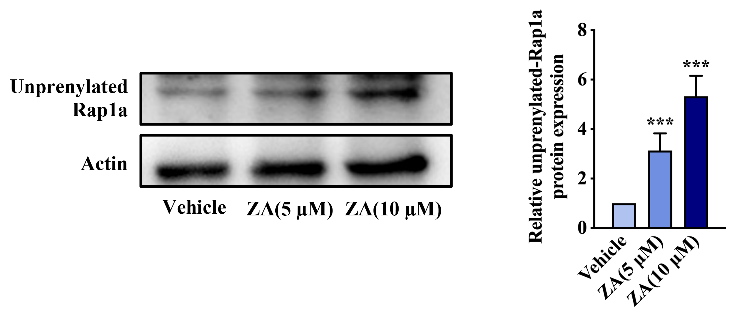


**Supplementary Figure 2.** ZA increase the level of unprenylated-Rap1a in BMDMs.

BMDMs were treated with vehicle or ZA for 24 h. The unprenylated Rap1a was detected using western blot. The data presented as the mean ± S.E.M. values (n=3). ********P*<0.001.
